# Pitfalls of anesthetic management with the Impella® 5.0 device: a case series

**DOI:** 10.1186/s40981-020-00324-9

**Published:** 2020-03-16

**Authors:** Naoshi Hotta, Akito Tsukinaga, Kenji Yoshitani, Yoshihiko Ohnishi

**Affiliations:** Department of Anesthesiology, National Cerebral and Cardiovascular Center, 6-1 Kishibeshinmachi, Suita, Osaka, 564-8565 Japan

**Keywords:** Percutaneous ventricular assist device, Advanced medical care, Heart failure

## Abstract

**Background:**

Impella® is an antegrade left ventricular assist device with a pump catheter in the left ventricle. We report three cases in which we experienced some pitfalls with circulatory management during Impella placement due to new-onset aortic insufficiency (AI) associated with device placement or the limited maximum flow rate.

**Case presentation:**

Three patients developed new-onset AI due to Impella placement. In a patient, the total assisted flow rate was relatively low because of his large body size. In the other patients, in whom the Impella device was used in combination with percutaneous cardiopulmonary support or venoarterial extracorporeal membrane oxygenation (ECMELLA), total flow was maintained at a sufficient level.

**Conclusions:**

New-onset of AI after Impella placement and its limited flow rate are considered to be pitfalls in circulatory management. Management with ECMELLA is considered to be effective during the acute phase when patients have decreased cardiac function.

## Background

Impella (Abiomed, Danvers, MA) is an antegrade left ventricular assist device (LVAD) with an intravascular microaxial blood pump that delivers blood from the left ventricle to the ascending aorta. It can be quickly placed with less invasiveness because placement does not require open-heart surgery. It can be used solely as an LVAD (LV-IMPELLA), a device for decompression of the left ventricle in combination with percutaneous cardiopulmonary support (PCPS) or venoarterial extracorporeal membrane oxygenation (VA ECMO) (ECMELLA) [1]. Clinical use of Impella was started in Europe in 2004 and in the USA in 2008, and it has been used in more than 50,000 patients all over the world. It has been available in the clinic since September 2016, and its use is spreading for patients with cardiogenic shock resistant to circulatory support by IABP and PCPS in Japan.

Among adverse events related to Impella, aortic insufficiency (AI) may impair hemodynamic conditions and should be carefully observed [2]. We report hemodynamic disturbance AI after insertion of Impella and limited flow rate of it in three cases.

## Case presentation

Table 1 shows the details of the three cases in which we found pitfalls with Impella 5.0 during anesthetic management. The primary diseases were ischemic cardiac myopathy, fulminant myocarditis, and dilated-phase of hypertrophic cardiomyopathy. PCPS and intra-aortic balloon pumping (IABP) were used in all patients for circulatory assistance. Due to insufficient organ perfusion and inadequate unloading of the failing left ventricle causing subsequent pulmonary congestion, Impella 5.0 was indicated. Impella 5.0 was placed under general anesthesia with intubation in all cases. Before entering the operating room, all these patients had been in sedative condition and intubated. Anesthesia was maintained with propofol (4–6 mg/kg/h), remifentanil (0.4–0.6 μg/kg/min), and rocuronium (0.4–0.5 mg/kg/h). Monitoring included electrocardiography, pulse oximetry, and transesophageal echocardiography. Data from an intra-arterial line, central venous catheter, and pulmonary artery catheter were also monitored. Under careful monitoring of hemodynamic conditions, IABP was ceased, and the catheter was withdrawn, followed by insertion of Impella 5.0 via subclavian artery. Patient 1 was switched from PCPS and IABP to LV-IMPELLA, and patients 2 and 3 were switched to ECMELLA with remaining PCPS. In three cases, the flow rate of Impella was not enough for viscera. Patients 2 and 3 required PCPS, but not patient 1. Then they weaned from PCPS within several days and switched to LV-IMPELLA.

**Table 1.** Circulatory parameters of the four patients

Patient	Age (years) /sex	BSA (m^2^)	Primary disease	Preoperative EF (%)	Preoperative concomitant VAD	ABP PAP CVP upon arrival in the operating room (after cessation of IABP) (mmHg)	Approach vessel	Assisted flow rate (L/min^-1^)	CCO (L/min) and CCI (L/min/m^2^) monitored with pulmonary artery catheter	Postoperative concomitant VAD	Total flow rate*^1^ (L/min) Total flow rate/BSA (L/min/m^2^)	Inotropic agent and vasoconstrictor dose*^2^ (μg/kg/min)	Mixed venous oxygen saturation (%)	ABP PAP CVP on postoperative day 1 (mmHg)	Duration of Impella 5.0 placement (days)	Outcome
1	58 M	1.7	ICM	11	PCPS IABP	94/58 (67) 32/19 (35) 7	RSCA	P-9 5.0	3.5 2.0	None	3.5 2.1	DOA 6 DOB 6 NAD 0.3	65%	64/59 (61) 44/26 (28) 5	8	Implantable LVAD
2	43 F	1.5	FMC	5-10	PCPS IABP	71/66 (66) 28/24 (26) 8	AsAo	P-9 4.5	3.1 2.1	PCPS 2.2 L/min	5.3 3.5	DOA 3 DOB 6	86%	86/74 (77) 19/11 (14) 10	17	Implantable LVAD
3	54 M	1.8	dHCM	10-15	PCPS IABP	70/52 (63) 42/28 (31) 12	RSCA	P-9 4.8	3.7 2.1	PCPS 2.1 L/min	5.8 3.2	DOA 3 NAD 0.1	70%	82/78 (79) 47/24 (33) 12	27	Implantable LVAD

*ICM* ischemic cardiomyopathy, *AMI* acute myocardial infarction, *FMC* fulminant myocarditis, *dHCM* dilated phase of hypertrophic cardiomyopathy, *BSA* body mass index, *PCPS* percutaneous cardiopulmonary support, *IABP* intra-aortic balloon pumping, *ECMO* extracorporeal membrane oxygenation, *ABP* arterial blood pressure, *PAP* pulmonary arterial pressure, *CVP* central venous pressure, *RSCA* right subclavian artery*, AsAo* ascending aorta, *CCO* continuous cardiac output, *CCI* continuous cardiac index, *AI* aortic insufficiency, *DOA* dopamine, *DOB* dobutamine, *NA* noradrenaline

*^1^Total flow rate is based on the sum of CCO determined with the thermodilution technique using the pulmonary artery catheter and PCPS flow rate

*^2^Maintenance dose after leaving the operating room

### New-onset aortic insufficiency due to Impella 5.0 placement

**Fig. 1:**
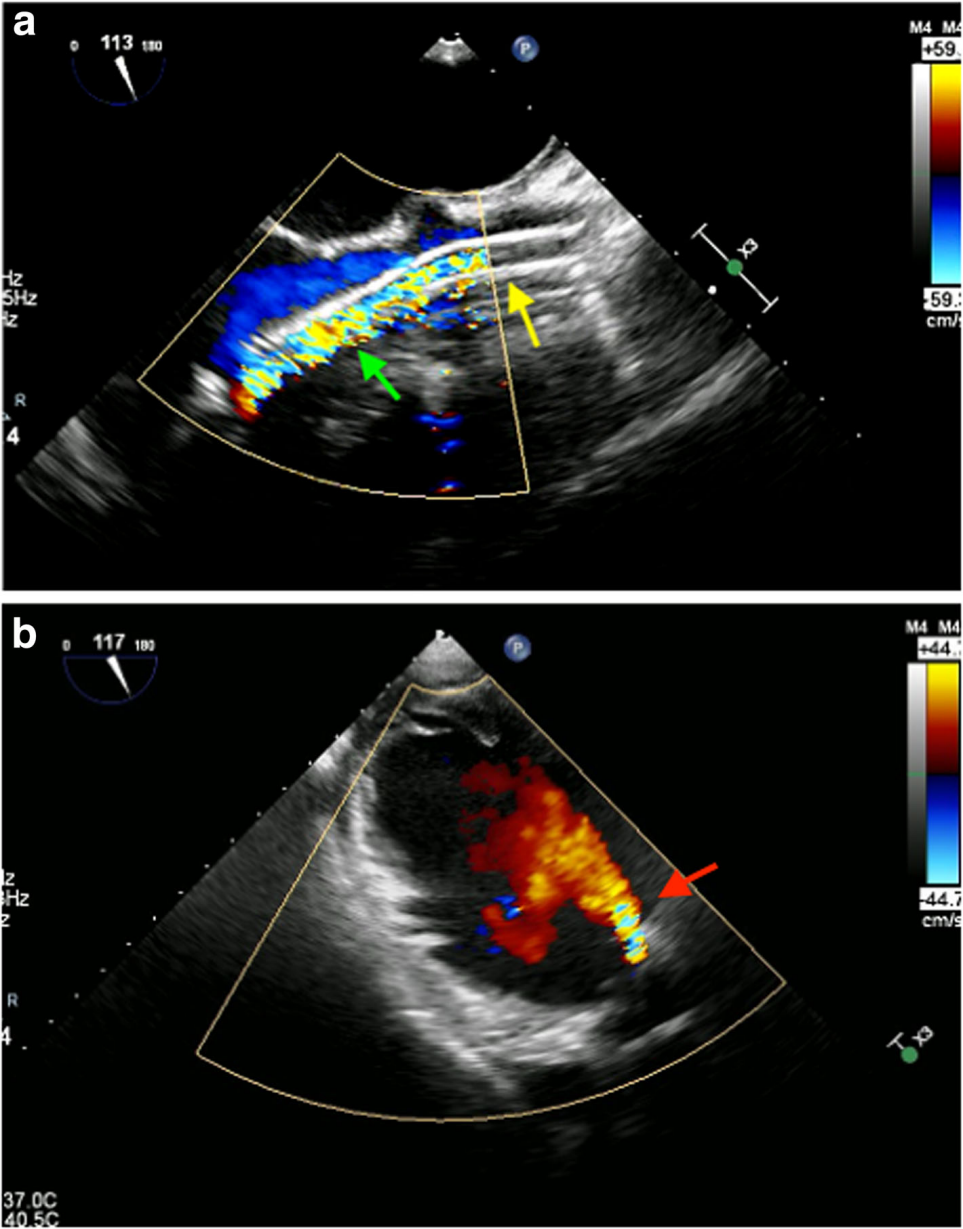
**a** Mid-esophageal long-axis view of transesophageal echocardiography. Yellow arrow indicates Impella cannula and green arrow indicates artifacts. Precise identification of aortic insufficiency by transesophageal echocardiography was difficult because of reverberation artifacts associated with axial blood flow inside of Impella. **b** Deep transgastric view of transesophageal echocardiography. Red arrow indicates regurgitation jet in the left ventricle due to aortic insufficiency. It was clearly identified without artifacts on this view

All patients developed new-onset aortic insufficiency and decreased flow rate after placement of Impella. In Patient 1, expected flow displayed on the Impella controller was 5.0 L/min with the maximum assistance level with Impella; however, continuous cardiac output (CCO) determined with the thermodilution technique using a pulmonary artery catheter was 3.5 L/min. TEE revealed newly developed AI after placement of Impella (Fig. 1a, b), suggesting that the decreased cardiac output by 1.5 L/min would correspond to the volume of AI. We suspected that AI was caused by incomplete leaflet coaptation resulting from non-perpendicular placement of Impella to the aortic valve. However, severity of AI was not decreased after adjusting its direction. Similarly, in patients 2 and 3, CCO was over 1 L/min lower than the expected flow displayed on the Impella controller, suggesting aortic insufficiency.

### Limited flow rate of the Impella 5.0 device

As shown in Table 1, the dose of noradrenaline was increased in all patients when they experienced a decrease in total assisted flow rate calculated as the sum of CCO and the flow of PCPS. In particular, the dose of noradrenaline in patient 1 was as high as 0.3 μg/kg/min.

In patient 1, pulse pressure was not observed due to an extremely low left ventricular ejection fraction (LVEF, 11%) even with the assisted flow of 5.0 L/min (maximum assistance level) with Impella. Furthermore, due to large body surface area (BSA, 1.7 m^2^) and new-onset aortic insufficiency, continuous cardiac index (CCI) remained at 2.0 L/min/m^2^, which was near the lower limit of normal. Large dose of noradrenaline (0.3 μg/kg/min) was required to maintain systemic blood pressure.

In patients 2 and 3, CCI remained at 2.1 L/min/m^2^, which was near the lower limit of normal, even though the Impella 5.0 assistance level was set to a maximum of P9. However, total flow rate was sufficient for organ perfusion from the point of absolute flow rate (5.3 L and 5.8 L) and flow rate corrected for body surface area (3.5 L/min/m2 and 3.2 L/min/m2) in patients 2 and 3, respectively, because the Impella 5.0 device was used in combination with PCPS as ECMELLA during the acute phase. Therefore, these patients did not require a dose of vasoconstrictors as high as in patient 1.

## Discussion

Impella 5.0 is an innovative ventricular assist device that can be placed in the heart quickly and with less invasiveness, but our experiences suggested that new-onset AI associated with placement of Impella and its limited flow rate could be pitfalls in circulatory management.

### New-onset aortic insufficiency

All three patients developed new-onset AI after Impella insertion. While a previous case report described a case of AI associated with aortic valve injury due to Impella insertion that persisted after removal [3], AI disappeared after removal of Impella, suggesting that the direct cause of AI was incomplete leaflet coaptation associated with mechanical compression by the cannula of Impella, rather than aortic valve injury in our cases.

Normal quantitative evaluation of AI during the use of a ventricular assist device may lead to underestimation because AI volume varies based on the duration of aortic valve closing. In some cases, AI may occur during the whole cardiac cycle [4]. In some patients without concomitant use of PCPS, such as patient 1, Impella placement may have a larger impact on hemodynamics. Therefore, we argue that AI should be considered in the differential diagnosis when a patient presents with low organ perfusion after Impella placement. In addition, when AI is visualized, artifacts associated with the Impella device also need to be taken into account [5]. For Patient 1, precise identification of AI by TEE was difficult because of reverberation artifacts associated with axial blood flow inside of Impella (Fig. 1a). It was clearly identified on the deep transgastric view (Fig. 1b), suggesting the requirement of careful observation for making a precise diagnosis.

### Limited flow rate of the Impella 5.0 device

In patient 1, for whom the Impella 5.0 device was used as LV-IMPELLA, the flow rate decreased both absolutely and relatively because new-onset AI and no spontaneous cardiac output passing through the aortic valve were observed during the whole cardiac cycle, including the diastolic and systolic phases, and organ perfusion decreased. A previous study also reported that LV-IMPELLA led to insufficient organ perfusion due to its limited flow rate including renal failure and liver failure [6]. Other LVADs with higher flow rates can compensate for reduced organ perfusion due to AI [7], and the BSA was relatively large. LV-IMPELLA may lead to insufficient organ perfusion in patients with LVEF that is too low to obtain spontaneous cardiac output. The impact of AI on hemodynamics becomes more significant because the maximum flow rate of the Impella 5.0 device is only 5.0 L/min and in patients with large BSA.

In patient 1, we could obtain flow rates that did not cause signs of circulatory failure such as increased lactate levels and decreased mixed venous oxygen saturation, but these flow rates were insufficient for maintaining normal blood pressure under anesthesia when systemic vascular resistance decreased. Therefore, with the use of the Impella 5.0 device, high-dose vasoconstrictor use is considered mandatory in some cases, as was the case with patient 1.

In patients 2 and 3, the Impella 5.0-assisted flow rate decreased because of new-onset AI. However, ECMELLA augmented the total flow rate and led to maintain arterial blood pressure and organ perfusion. Therefore, only a small dose of vasoconstrictors (or none at all) was required to maintain systemic blood pressure. In a previous case study, a patient in the acute phase of fulminant myocarditis with progressive organ failure during LV-IMPELLA use improved organ function after that Impella 5.0 device was used as ECMELLA in combination with PCPS [8]. Management with ECMELLA was considered to be effective when LV-IMPELLA does not improve circulatory failure due to the limited flow rate. In patients 2 and 3, spontaneous cardiac output started after improvement in cardiac function, and AI was limited to the diastolic phase, allowing for switching from ECMELLA to LV-IMPELLA.

## Conclusions

New-onset of AI after Impella 5.0 placement and its limited flow rate are considered to be pitfalls in circulatory management. When the anesthesiologists encountered AI after placement of Impella, ECMELLA is considered to be effective during the acute phase until patients improve cardiac function.

### Ackowledgements

None

## Data Availability

Not applicable

## Abbreviations

AI: Aortic insufficiency
CCI: Continuous cardiac index
CCO: Continuous cardiac output
IABP: Intra-aortic balloon pumping
LVAD: Left ventricular assist devise
LVEF: Left ventricular ejection fraction
PCPS: Percutaneous cardiopulmonary support
VA ECMO: Venoarterial extra-corporeal membranous oxygenation
